# Association Analysis of *IL10, TNF*-α, and *IL23R*-*IL12RB2* SNPs with Behçet’s Disease Risk in Western Algeria

**DOI:** 10.3389/fimmu.2013.00342

**Published:** 2013-10-21

**Authors:** Ouahiba Khaib Dit Naib, Mourad Aribi, Aicha Idder, Amel Chiali, Hakim Sairi, Isabelle Touitou, Gérard Lefranc, Mouna Barat-Houari

**Affiliations:** ^1^Laboratory of Applied Molecular Biology and Immunology. Department of Biology, Abou-Bekr Belkaïd University, Tlemcen, Algeria; ^2^Departments of Pharmacy and Oral Medicine, Abou-Bekr Belkaïd University, Tlemcen, Algeria; ^3^Etablissement Hospitalier Spécialisé en Ophtalmologie, Clinique Hamou Boutlelis, Oran, Algeria; ^4^Service de Dermatologie, Centre Hospitalo-Universitaire d’Oran, Oran, Algeria; ^5^Unité Médicale des Maladies Auto-inflammatoires, Département de Génétique, CHRU, Montpellier, France; ^6^Université Montpellier 1, Montpellier, France; ^7^Génétique des Maladies Auto-inflammatoires et des Ostéo-arthropathies chroniques, INSERM U844, Montpellier, France; ^8^Laboratoire d’Immunogénétique Moléculaire, Institut de Génétique Humaine, CNRS UPR 1142, et Université Montpellier 2, Montpellier, France

**Keywords:** Behçet’s disease, genetic association, *IL10*, *TNF*-α, *IL23R*-*IL12RB2*, single nucleotide polymorphism, Western Algeria

## Abstract

**Objective:** We have conducted the first study of the association of interleukin (IL)-10, tumor necrosis factor alpha (*TNF*-α), and *IL23R*-*IL12RB2* region single nucleotide polymorphisms (SNPs) with Behçet’s disease (BD) in Western Algeria.

**Methods:** A total of 51 BD patients and 96 unrelated controls from West region of Algeria were genotyped by direct sequencing for 11 SNPs including 2 SNPs from the *IL10* promoter [c.-819T > C (rs1800871), c.-592A > C (rs1800872)], 6 SNPs from the *TNF*-α promoter [c.-1211T > C (rs1799964), c.-1043C > A (rs1800630), c.-1037C > T (rs1799724), c.-556G > A (rs1800750), c.-488G > A (rs1800629), and c.-418G > A (rs361525)], and 3 SNPs from the *IL23R*-*IL12RB2* region [g.67747415A > C (rs12119179), g.67740092G > A (rs11209032), and g.67760140T > C (rs924080)].

**Results:** The minor alleles c.-819T and c.-592A were significantly associated with BD [odds ratio (OR) = 2.18; 95% confidence interval (CI) 1.28–3.73, *p* = 0.003]; whereas, there was weaker association between *TNF*-α promoter SNPs or *IL23R*-*IL12RB2* region and disease risk.

**Conclusion:** Unlike the *TNF*-α and the *IL23R*-*IL12RB2* region SNPs, the two *IL10* SNPs were strongly associated with BD. The -819T, and -592A alleles and the -819TT, -819CT, and -592AA and -592CA genotypes seem to be highly involved in the risk of developing of BD in the population of Western Algeria.

## Introduction

Behçet’s disease (BD) is a systemic inflammatory multifactorial disease (1). It is characterized by recurrent episodes of oral and genital ulceration, skin, and ocular lesions (2). The disease is now recognized as a systemic vasculitis, given that it can affect other tissues and organs including blood vessels, the digestive tract, and nervous system (3, 4).

The etiology of BD is not fully elucidated. There is a hypothesis that a pathogenic autoimmune process of BD is triggered by an infectious or environmental agent, in individuals genetically predisposed (5). The most strongly genetic factor associated with the disease is HLA B51 (5, 6). This association was initially described in 1973 (7) and subsequently confirmed in different ethnic groups (6, 8, 9).

However, the association between BD and HLA B51 represents only 20% in the siblings of patients with the disease (10), and 50% of cases with BD are negative for this allele (8, 9). These observations suggest the existence of other risk factors outside the HLA region. In fact, several recent genome-wide association studies have identified additional and new genomic regions that predispose to the disease (11–14).

The BD causes inflammation and chronic immune activation within small blood vessels (15–17). The site of inflammation is usually characterized by infiltration of immune cells as well as by highly elevated levels of different cytokines (18). IL-10 is one of the most important cytokines that has been observed at increased level in the serum and active lesions of BD patients (19–21). TNF-α and soluble TNF-α receptors are also elevated in the sera of patients with BD (22–24). Both IL-10 and TNF-α cytokines have been shown to play an important role in the immunopathology of autoimmune diseases (25–28), and an opposite roles in the inflammatory responses (29, 30). An autoregulatory loop appears to exist in whereby TNF-α induces IL-10 production, which ultimately reduces TNF-α synthesis (31, 32). It has been suggested that the increase of IL-10 may down-regulate the expression of NO, prompting the protective role of elevation of IL-10 (33). Additionally, treatment with anti-TNF-α monoclonal antibodies has resulted in improvement of various manifestations of BD (34, 35).

IL-10 and TNF-α production may be regulated at the transcriptional level. Thus, several single nucleotide polymorphisms (SNPs) at the promoter of *IL10* and *TNF*-α gene have been shown to be associated with changes in the expression levels of IL-10 and TNF-α production (36, 37). On the other hand, numerous recent studies have demonstrated an association between BD and several *IL10* (13, 14, 38, 39) and *TNF*-α (39–43) SNPs in different ethnic groups. However, to date, there are no analogous or identical investigations in Algeria.

Two others cytokines, IL-23 and IL-12, may play an important role in BD pathogenesis; their levels are elevated in BD patients (44–46). IL-23 drives and promotes the development of a unique T-helper cell population that produces IL-17, Th17 cells. These IL-23-driven Th17 cells are highly pathogenic and elicit IL-17-dependent inflammation in autoimmune diseases (47). IL-12, a heterodimeric cytokine, is of crucial relevance to cell-mediated immunity and Th1 differentiation (48). This cytokine exerts its biological effects *via* binding to a heterodimeric receptor consisting of IL12RB2 and IL-12RB1 subunits.

The effect of IL-23 and IL-12 is mediated through the IL-23 and the IL-12 receptor (IL-23R, IL-12RB1). The genes that encode these receptors are adjacent on chromosome 1p31; a GWAS Studies revealed that *IL23R*-*IL12RB2* region is associated with BD (13, 14). Nevertheless, its association in the pathogenesis of BD remains to be confirmed in different ethnic groups. In this context, we examined genetic association for 11 SNPs in *IL10, TNF*-α, and *IL23R*-*IL12RB2* candidate genes with BD in Western Algeria.

## Materials and Methods

### Patients and subjects

Fifty-one (51) unrelated BD patients and age- and sex-matched 96 healthy controls originate from the Western Algeria were recruited for a case-control study at the Oran Ophthalmic Hamou Boutlelis Hospital, the Department of Dermatology of Oran Medical Centre University, and the Oran Blood Transfusion Centre (Algeria). Among the 51 patients, 11 DNA belonging to Algerian origin, were selected from the biobank DNA for Genetics Laboratory of Autoinflammatory Diseases, Arnaud de Villeneuve Hospital, Montpellier (France).

Consent was signed by each participant or participant’s parent or legal guardian if entrant is a minor, under the Rules of Ethics and Professional Conduct. Patient characteristics were recorded using a questionnaire. The diagnosis of patients was based especially on the criteria proposed in 1990 (49). The control group was composed of healthy subjects without a family history of autoinflammatory diseases, and selected from the same population. This work was approved by the Institutional Ethics Board of Tlemcen Abou-Bekr Belkaïd University.

### Genotyping

Each DNA was genotyped for 11 SNPs, including two *IL10* promoter SNPs [c.-819T > C (rs1800871), c.-592A > C (rs1800872)], six SNPs from the *TNF*-α promoter [c.-1211T > C (rs1799964), c.-1043C > A (rs1800630), c.-1037C > T (rs1799724), c.-556G > A (rs1800750), c.-488G > A (rs1800629), and c.-418G > A (rs361525)], and three SNPs from the *IL23R*-*IL12RB2* region [g.67747415A > C (rs12119179), g.67740092G > A (rs11209032), and g.67760140T > C (rs924080)].

Genotyping was performed at the Laboratory of Genetics of Autoinflammatory Diseases, Arnaud de Villeneuve Hospital, Montpellier (France). Genomic DNA was isolated from peripheral blood, drawed on EDTA anti-coagulant, using QIAamp DNA Blood Kits (Qiagen, Valencia, CA, USA). The DNA samples were then dosed by spectrophotometry ND-1000 (Nano Drop Technologies, Wilmington, DE, USA) at 260 and 280 nm. The DNA concentration and ratio OD260/OD280 were estimated for each sample (50).

The DNA samples were subsequently amplified in a Applied Biosystems Thermocycler (Applied Biosystems, Foster City, CA, USA) in a 15 μL reaction volume containing 50 ng DNA, 2X Promega PCR Master Mix, and 25 μM of each primer (Table 1). The PCR programs were as follows: after a denaturation phase of 15 min at 95°C, the samples were subjected to 35 amplification cycles followed by a final elongation step of 7 min at 72°C. Each cycle comprises 30 s denaturation at 95°C, 30 s of primer annealing at 60°C, and 1 min extension at 72°C.

**Table 1:** **Primers sequence and length product**.

Loci	SNPs	Forward primer	Reverse primer	Product length (bp)
*IL10*	rs1800871 rs1800872	TTAGACTCCAGCCACAGAAGC	GGGGGACCCAATTATTTCTC	597
*TNF-α*	rs1799964 rs1800630 rs1799724	GTGTGTCTGGGAGTGAGAACTTC	CTTCTTTCATTCTGACCCGG	570
	rs1800750 rs1800629 rs611525	CTCAGGACTCAACACAGCTTTTC	GAAAGAATCATTCAACCAGCG	438
*IL23R-IL12RB2* region	rs11209032	GGAGTTAAACCTCTTGCTATCCTG	GATGCACAATGAGTTGATAAGG	164
	rs12119179	TACCCAGGGCATTCAGCTAC	GCTTGAGCTCCTGGATCAAG	701
	rs924080	GCACGTATGCCTTTTTGCATA	ATTTGAATGTGCCTTGGCAT	364

*bp, base pair; IL, interleukin; rs, reference SNP; SNP, single nucleotide polymorphism; TNF, tumor necrosis factor*.

**Figure 1:**
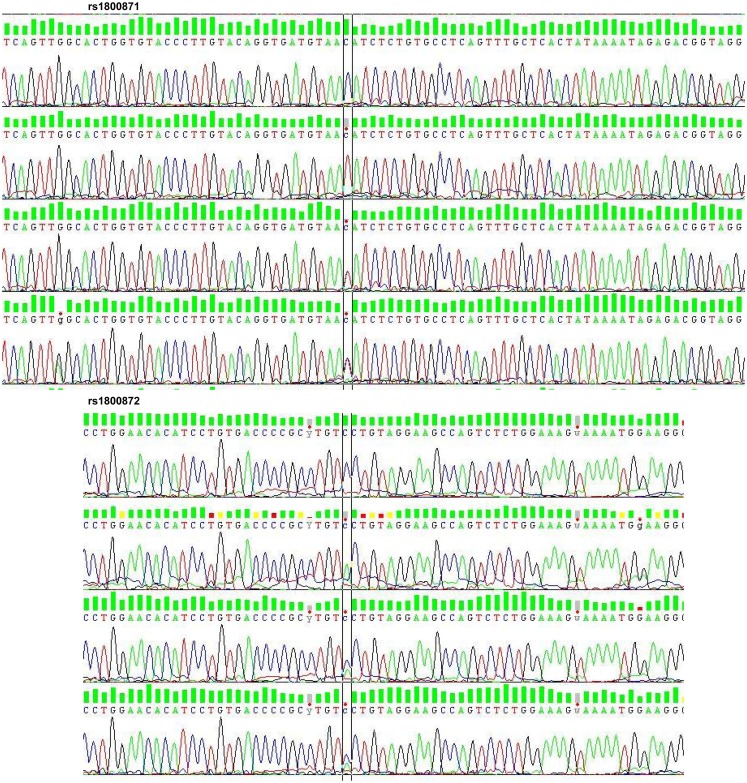
**Electropherogram of rs1800871 and rs1800872**. rs, reference SNP; SNP, single nucleotide polymorphism.

After checking the quality and size of the PCR products by agarose gel (1.5%) electrophoresis, SNPs genotyping was performed by direct sequencing using the BigDye Terminator version 3.1 (BDT v3.1) Cycle Sequencing Kit, followed by capillary electrophoresis on an ABI 3100XL Genetic Analyzer, according to the manufacturer’s recommendations (Applied Biosystems, Foster City, CA, USA) (Figure 1).

### Statistical analysis

Comparisons of allele and genotype frequencies between groups (patients versus control subjects, and between the patient’s groups according to different clinical features) were performed using the Chi-square or Fisher’s exact tests. The association analysis was carried out by Odds ratio (OR) and corresponding 95% confidence interval (95% CI). Statistical analyses were performed using GraphPad Prism Version 5.04 (GraphPad Software, Inc., La Jolla, CA, USA) and Epi Info 2000 Version 1.0 for Windows (Epi Info, Atlanta, GA, USA) software.

## Results

Table 2 shows the description of the clinical characteristics of the patients with BD of the current study. The mean age (±SD) of the patients at disease onset was 26 ± 11 years. Predominant lesions were oral ulcers (100%), cutaneous lesions (86.27%), genital ulcers (82.35%), eye lesions (62.74%), and arthritis (58.82%).

**Table 2:** **Clinical and demographic features of the Behçet patients of the current study**.

Characteristics	Frequency (*n* = 51)
Mean age at disease onset ± SD (year)	26 ± 11
Sex ratio M/F (%, *n*)	56.9/43.1 (29/22)
Oral ulcers (%, *n*)	100 (51)
Genital ulcers (%, *n*)	82.4 (42)
Cutaneous lesions (%, *n*)	86.3 (44)
Eye lesions (%, *n*)	62.7 (32)
Neurological symptoms (%, *n*)	35.3 (18)
Venous thrombosis (%, *n*)	25.5 (13)
Arthritis (%, *n*)	58.8 (30)
Multiplex family (%, *n*)	35.3 (18)
pediatric case (%, *n*)	19.6 (10)
consanguinity (%, *n*)	43.1 (22)

*SD, standard deviation*.

The distribution of alleles and genotypes frequencies of *IL10* promoter SNPs c.-819C > T (rs1800871) and c.-592C > A (rs1800872) showed that the two SNPs were in total linkage disequilibrium in our sample. For this, reason the results of one SNP c.-819C > T will be considered (Table 3).

**Table 3:** **Allelic and genotypic frequencies of rs1800871 variant in BD patients and controls**.

Alleles and genotypes	Frequencies (%)		*p*
	Controls (*n* = 96)	Cases (*n* = 51)	
C	141 (73.4)	57 (55.9)	0.003**
T	51 (26.6)	45 (44.1)	
CC	50 (52.1)	17 (33.3)	0.005**
CT	41 (42.7)	23 (45.1)	
TT	5 (5.2)	11 (21.6)	

BD, Behçet’s disease; SNP, single nucleotide polymorphism; rs, reference SNP. **p < 0.01.

**Figure 2:**
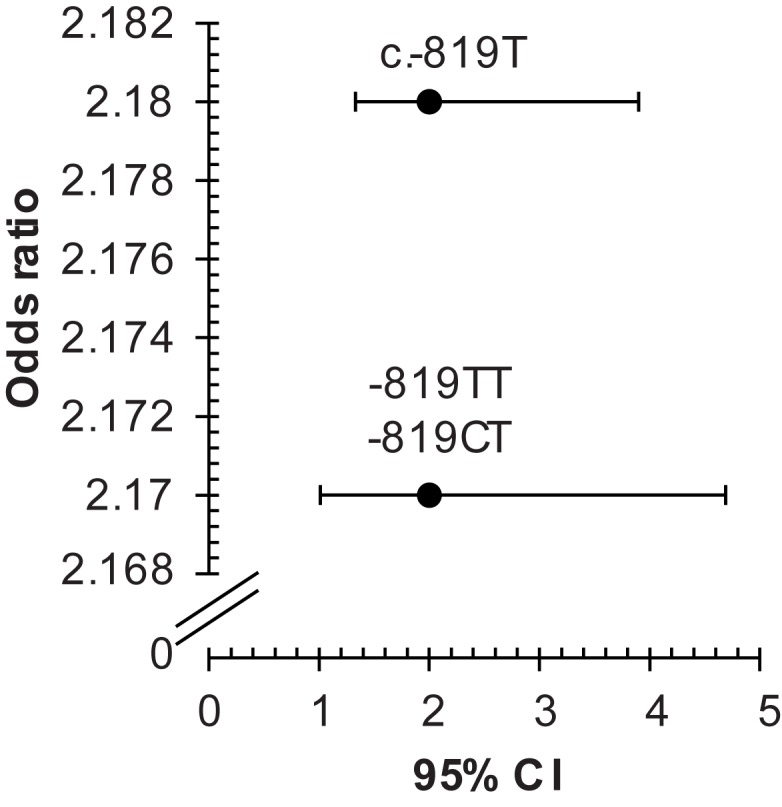
**Odds ratios for associations between *IL10* c.-819C > T, and -819TT, -819CT (rs1800871) with Behçet’s disease**. The two SNPs c.-819C > T and c.-592C > A are in total linkage disequilibrium in our sample; therefore, the results of only one SNP c.-819C > T is considered. CI, confidence interval; rs, reference SNP; SNP, single nucleotide polymorphism.

The allele frequencies were significantly different in patients compared to controls. As indicated in Table 3, the frequencies of c.-819T (rs1800871) allele, and of the -819TT, -819CT (rs1800871) genotypes were significantly increased in patients than in controls (*p* = 0.003 and *p* = 0.005, respectively). Additionally, these SNPs was significantly associated with the disease (c.-819T; OR = 2.18, 95% CI 1.28–3.73, *p* < 0.01; -819TT and -819CT, OR = 2.17, 95% CI 1.01–4.69, *p* < 0.05) (Figure 2).

A subset analysis was performed to examine the difference in allele frequencies in clinical subsets of BD (Table 4). We observed a significant association between c.-819T and all classes; nevertheless, the association was slightly lower for the ocular lesion (OR = 1.55, 95% CI 0.81–2.96, *p* > 0.05). Additionally, the association was more significant for the Genital ulcers (OR = 2.21; 95% CI 1.29–4.04, *p* = 0.002).

**Table 4:** **Association analysis of clinical subclasses with *IL10* c.-819T SNP in patients with Behçet’s disease**.

Clinical subset	OR	95% CI		*p*
		LL	UL	
Eye disease	1.55	0.81	2.96	0.152
Genital ulcers	2.21	1.29	4.04	0.002**
Skin lesions	2.07	1.17	3.68	0.007**
Arthritis-arthralgia	2.06	1.11	3.81	0.013*
Neurologic signs	2.5	1.18	5.32	0.009**

CI, confidence interval; LL, lower limit; UP, upper limit; OR, odds ratio; SNP, single nucleotide polymorphism. *p < 0.05, **p < 0.01.

We reported in Tables 5 and 6 that all *IL23R*-*IL12RB2* SNPs alleles and genotypes, respectively, were not significantly associated with the disease (OR > 1, *p* > 0.05). The minor allele frequencies were different in the two groups, but this difference did not reach statistical significance (*p* > 0.05).

**Table 5:** ***IL23R*-*IL12RB2* and *TNF*-α allelic frequencies in patients with Behçet’s disease**.

Loci	SNPs	Alleles frequency (proportion, %)		OR (95% CI)	*p*
*IL23R*-*IL12RB2*	rs12119179 (g.67747415A > C)	A	C	1.24 (0.72–2.13)	0.415
	Patients	67 (65.7)	35 (34.3)	
	Controls	135 (70.3)	57 (29.7)	
	rs11209032 (g.67740092G > A)	G	A	1.18 (0.68–2.03)	0.530
	Patients	67 (65.7)	35 (34.3)	
	Controls	133 (69.3)	59 (30.7)	
	rs924080 (g.67760140T > C)	T	C	1.45 (0.87–2.41)	0.133
	Patients	54 (52.9)	48 (47.1)	
	Controls	84 (43.8)	108 (56.3)	
*TNF*-α	rs1799964 (c.-1211T > C)	T	C	0.92 (0.52–1.61)	0.751
	Patients	73 (71.6)	29 (28.4)	
	Controls	134 (69.8)	58 (30.2)	
	rs1800630 (c.-1043C > A)	C	A	0.83 (0.41–1.67)	0.584
	Patients	86 (84.3)	16 (15.7)	
	Controls	157 (81.8)	35 (18.2)	
	rs17999724 (c.-1037C > T)	C	T	1.01 (0.35–2.84)	0.976
	Patients	95 (93.1)	7 (6.9)	
	Controls	179 (93.3)	13 (6.7)	
	rs1800750 (c.-556G > A)	G	A	0.61 (0.14–2.09)	0.402
	Patients	98 (96.1)	4 (3.9)	
	Controls	180 (93.7)	12 (6.3)	
	rs1800629 (c.-488G > A)	G	A	1.12 (0.56–2.26)	0.726
	Patients	85 (83.3)	17 (16.7)	
	Controls	163 (84.9)	29 (15.1)	
	rs361525 (c.-418G > A)	G	A	0.93 (0.39–2.21)	0.869
	Patients	92 (90.2)	10 (9.8)	
	Controls	172 (89.6)	20 (10.4)	

*CI, confidence interval; *IL23R*-*IL12RB2*, region of the interleukin-23 receptor and interleukin-12 receptor beta 2 chain-encoding gene; OR, odds ratio; rs, reference SNP; SNP, single nucleotide polymorphism; TNF, tumor necrosis factor*.

**Table 6:** **The distribution of *IL23R*-*IL12RB2* genotypes in patients with Behçet disease**.

SNPs	Genotype distribution (frequency, %)			*p*-Value	OR (95% CI) *p*-value	MAF (%)	OR^MAF^ (95% CI) *p*-value
rs12119179 (g.67747415A > C)	AA	AC	CC	0.522	1.13 (0.54–2.35) 0.734	C	1.24 (0.58–2.67) 0.580
Patients	24 (47.1)	19 (37.2)	8 (15.7)			34.31	
Controls	48 (50)	39 (40.6)	9 (9.4)			29.69	
rs11209032 (g.67740092G > A)	GG	GA	AA	0.721	1.12 (0.57–2.21) 0.744	A	1.18 (0.71–1.97) 0.560
Patients	23 (45.1)	21 (41.2)	7 (13.7)			34.31	
Controls	46 (47.9)	41 (42.7)	9 (9.4)			30.73	
rs924080 (g.67760140T > C)	CC	CT	TT	0.327	1.46 (0.64–3.35) 0.326	C	1.58 (0.71–3.54) 0.264
Patients	13 (25.5)	22 (43.1)	16 (31.4)			47.1	
Controls	32 (33.4)	44 (45.8)	20 (20.8)			56.25	

*ORs were calculated for the minor versus major alleles. CI, confidence interval; *IL23R*-*IL12RB2*, interleukin-23 receptor and interleukin-12 receptor beta 2 chain-encoding gene; MAF, minor allele frequency; OR^MAF^, OR of the MAF; OR, odds ratio; rs, reference SNP; SNP, single nucleotide polymorphism*.

As indicated in Tables 5 and 7, alleles and genotypes of the *TNF*-α polymorphisms display similar distributions in patients and controls (*p* > 0.05). Except for c.-1037T and c.-488A all others *TNF*-α alleles were not associated with BD (OR < 1).

**Table 7:** **The distribution of *TNF*-α genotypes in patients with Behçet disease**.

SNPs	Genotype distribution (frequency, %)			*p*-Value	OR (95% CI) *p*-value	MAF (%)	OR^MAF^ (95% CI) *p*-value
rs1799964 (c.-1211T > C)	TT	TC	CC	0.114	0.67 (0.32–1.4) 0.249	C	0.8 (0.37–1.75) 0.580
Patients	29 (56.9)	15 (29.4)	7 (13.7)			28.43	
Controls	45 (46.9)	44 (45.8)	7 (7.29)			30.21	
rs1800630 (c.-1043C > A)	CC	CA	AA	0.826	0.79 (0.35–1.78) 0.544	A	0.78 (0.31–1.97) 0.600
Patients	37 (72.5)	12 (23.5)	2 (4)			15.69	
Controls	65 (67.7)	27 (28.1)	4 (4.2)			18.23	
rs17999724 (c.-1037C > T)	CC	CT	TT	ND	1.23 (0.4–3.74) 0.690	T	1.26 (0.34–4.69) 0.733
Patients	44 (86.3)	7 (13.7)	0 (0)			6.86	
Controls	83 (86.5)	13 (13.5)	0 (0)			6.77	
rs1800750 (c.-556G > A)	GG	GA	AA	ND	0.6 (0.13–2.12) 0.388	A	0.6 (0.12–3.07) 0.536
Patients	47 (92.2)	4 (7.8)	0 (0)			3.92	
Controls	84 (87.5)	12 (12.5)	0 (0)			6.25	
rs1800629 (c.-488G > A)	GG	GA	AA	0.909	1.17 (0.52–2.61) 0.680	A	1.18 (0.47–2.97) 0.721
Patients	35 (68.6)	15 (29.4)	1 (2)			16.67	
Controls	69 (71.9)	25 (26)	2 (2.1)			15.1	
rs361525 (c.-418G > A)	GG	GA	AA	0.299	0.75 (0.28–2.02) 0.541	A	0.9 (0.29–2.79) 0.849
Patients	43 (84.3)	6 (11.8)	2 (3.9)			9.8	
Controls	77 (80.2)	18 (18.8)	1 (1)			10.42	

*ORs were calculated for the minor versus major alleles. CI, confidence interval; MAF, minor allele frequency; ND, not defined; OR, odds ratio; OR^MAF^, OR of the MAF; rs, reference SNP; SNP, single nucleotide polymorphism; TNF, tumor necrosis factor*.

## Discussion

To date, the etiopathogenesis of BD is not fully elucidated. Researches in recent decades have shown the complex role of genetic factors in the development of the disease. We analyze the association between BD and 11 SNPs in *IL10, TNF*-α, and *IL23R*-*IL12RB2* candidate genes in the Western Algeria population.

This is the first report demonstrating that the c.-819T and c.-592A alleles were associated with BD in Algeria.

Previous genetic studies have shown a strong association of many *IL10* variants with BD in different ethnic groups. Recent genome-wide association study conducted by Mizuki et al. (14) in a Japanese cohort, including 612 individuals with BD and 740 unaffected individuals controls from different ethnic groups, has shown a significant difference between the two groups for five *IL10* SNPs. The two SNPs analyzed in our study showed an association (OR = 2.18, 95% CI 1.28–3.73, *p* < 0.01). On the other hand, a strong association has also been highlighted for *IL10* rs1518111 (OR = 1.45, 95% CI 1.34–1.58) in a genome-wide study performed in 311,459 SNPs in 1215 individuals with BD and 1278 healthy controls from Turkey (13). Our results are consistent with those obtained by Wallace et al. (38) who studied the segregation of two *IL10* promoter SNPs, rs1800871 and rs1800896 in 178 cases and 295 controls from two populations, including Arab Middle East and United Kingdom. A strong association of the 819T allele has been observed in United Kingdom patients (OR 1.5, 95% CI 1.1–2).

Our results are, however, in disagreement with those of the study of Ates et al. (39) in which no significant association was revealed by exploring three *IL10* SNPs (-1082G > A, rs1800896, c.-819C > T, rs1800871, and c.-592C > A, rs1800872) in 102 patients with BD and 102 controls from Turkey. These conflicting results may be explained by ethnic differences.

Several studies have shown the association of the two (c.-819 C > T, c.-592C > A) studied SNPs with various inflammatory diseases, but also with cancer (51–53), periodontitis (54), and docetaxel-induced liver injury (55). These associations suggested that the two SNPs can play an important role in the expression of *IL10*. In fact, it has been previously reported that the SNP c.-819C > T and/or c.-592C > A alleles affect the transcription of *IL10* (56). Other studies performed on three SNPs at position -1082 A > G, -819C > T, and -592C > A in the promoter region of the *IL10* gene have shown that the expression levels of IL-10 was significantly different according to the some haplotypes (57, 58). Finally, it has been reported that the disease-associated rs1518111 allele seems to be associated with low IL-10 mRNA expression and protein production (13). Indeed at the SNP rs1518111 locus, the rate of transcript of the G allele is higher than that of the A allele and patients homozygous for the A allele produce less IL-10 than those who are heterozygous or homozygous for the G allele.

The result of subset analysis suggests that the risk allele might predispose to genital ulcers, skin lesions, neurologic signs, and arthritis-arthralgia, but weakly to eye complications; the association was more significant for the genital ulcers (OR = 2.21, *p* = 0.002). Our results are in agreement with those of previous study (21) that showed a variable increase in mRNA expression within all BD lesions, including oral and genital ulcers, pseudofolliculitis lesions, and lesions at the site of pathergy testing.

Recent GWAS study from Turkey and Japan revealed *IL12R*-*IL23RB2* SNPs in association with BD. Three SNPs were strongly associated with the disease, including rs924080 (OR = 1.28, *p* = 6.69 × 10^−9^) (13), rs12119179 (*p* = 2.7 × 10^−8^), and rs1495965 (OR = 1.35, *p* = 1.9 × 10^−11^) (14), but no significant association was found in a Korean cohort.

Our results showed no significant association between BD and rs12119179, g.67740092G > A (rs11209032), and g.67760140T > C (rs924080) SNPs in the *IL23R*-*IL12RB2* region. In Iranian study (59), six SNPs in *IL23R*-*IL12RB2* were found to be associated with BD; the most significant of which were rs17375018 (OR = 1.51, *p* = 1.93 × 10^−6^), rs7517847 (OR = 1.48, *p* = 1.23 × 10^−6^), and rs924080 (OR = 1.29, *p* = 1.78 × 10^−5^). Others studies have also identified a strong relationship between polymorphisms of *IL23R* and BD (60–62). These associations may suggest an important role of Th17 cells that express the IL-23R on their surface. Kim et al. (62) studied the interaction of specific *IL17A, IL23R*, and STAT4 (signal transducers and activators of transcription 4) SNPs in intestinal BD Korean patients; they suggest that the IL-23/IL-17 axis plays a significant role in disease pathogenesis.

IL-12 has been implicated in the pathogenesis of a multitude of diverse autoimmune diseases (63, 64). *IL12RB2* constitute a risk factor for primary biliary cirrhosis, with the reported top associated SNPs mainly located in intronic sequences (65–67).

The genetic architecture and modularity of human autoimmune diseases is very complex. The functional implications of most of these associations are not yet clarified. Identify candidate causal SNPs and pathways (ICSN Pathway) analysis may act as a powerful guide to further research into the functional and immunological ramifications of these associations.

No significant associations were found between BD and studied *TNF*-α polymorphisms. These SNPs have been studied in various ethnic groups for possible association with BD. However, the allelic and genotypic associations of these studies have been contradictory. In Korean patients, *TNF*-α c.-1043A (rs1800630) allele was associated with an increased risk of BD (OR = 1.4, *p* = 0.030) (68). However, no significant association was found in meta-analysis studies for this SNP (42). Additionally, it has been reported a significant associations between c.-1037T allele (rs1799724) (OR = 0.76, 95% CI 0.58–0.98), c.-488G allele (rs1800629) (OR = 1.8, *p* = 0.010) (68), and c.-418A allele (rs361525) (OR = 1.51, 95% CI 1.12–2.04) (42), and BD. Moreover, no significant associations were identified with other *TNF*-α promoter polymorphisms, such as c.-1037T (rs1799724), c.-488A (rs1800629), and c.-556G > A (rs1800750) alleles with BD in Moroccan patients (43). The *TNF*-α c.-1211C allele (rs1799964) presented a significant association with BD in several populations, including Turkish (*p* = 0.023) (69), Korean (*p* = 0.030, OR = 1.4) (68), and UK white Caucasoid population (RR = 2.3, *p* = 0.00004) (40). The frequency of the *TNF*-α c.-1211C allele was significantly higher in Behcet’s patients than in healthy controls in Moroccan and Tunisian populations (OR = 1.65, *p* = 0.015; OR = 1.68, *p* = 0.02, respectively) (41, 43) and in meta-analysis (OR = 1.35, 95% CI 1.09–1.68) (42). This polymorphism has been associated with several extra-intestinal manifestations of Crohn’s disease, including uveitis, erythema nodosum, and large joint arthropathy (70), all of which are known to be associated with BD. Further investigation is necessary to determine the functional significance of *TNF*-α c.-1691042C and how it participates in the inflammatory dysregulation associated with BD.

Thus, polymorphisms at positions c.-1211T > C, c.-1043C > A, c.-1037C > T, and c.-488G > A have been associated with increased transcriptional activity and production of TNF-α in some studies (37, 71), in contrast to others (72–75). The over production of TNF-α during the course of BD may result in other *TNF*-α polymorphisms or post-transcriptional mechanisms. Furthermore, TNF-α production is not only under the control of the promoter region of *TNF*-α, and it may also result from complex cis and trans interactions among other cytokines.

*TNF*-α is encoded in the HLA complex on chromosome 6, a region that has long been known to be associated with BD. This gene-dense region, presentes a strong linkage disequilibrium (76). The association between BD and *TNF*-α could therefore be a result of linkage disequilibrium with alleles within this group. So it will be interesting to investigate other genes polymorphism among this region in our population.

In conclusion, we replicate the associations between BD and the SNPs from the *IL23R*-*IL12RB2* region and c.-1037C > T and c.-488G > A *TNF*-α promoter SNPs. *IL10* promoter SNPs (rs1800871 and rs1800872) is strongly associated with BD in the population of the Western Algeria. It would be interesting to study other SNPs to identify additional associations with BD in the studied population.

## Authors Contribution

Mourad Aribi, Gérard Lefranc are Principal Investigators of the study, participated in its design and execution and helped draft the manuscript and critically reviewed it for intellectual content; Mouna Barat-Houari participated in the design of the study, carried out genetic analyses, and helped draft the manuscript; Ouahiba Khaib Dit Naib wrote the manuscript and carried out genetic analyses; Aicha Idder, Amel Chiali, and Hakim Sairi are responsible for the recruitment of eligible patients and their families; Isabelle Touitou conceived of the study, participated in its design, and coordination. All the authors read and approved the final manuscript.

## Conflict of Interest Statement

The authors declare that the research was conducted in the absence of any commercial or financial relationships that could be construed as a potential conflict of interest.
